# Sarcoidosis of the pineal region, dorsal mesencephalon, and periaqueductal region: Case report and systematic review

**DOI:** 10.5339/qmj.2021.29

**Published:** 2021-08-23

**Authors:** Khelifa Adel, Walid Bennabi, Fayçal Aichaoui, Salim Meziani, Abdelhalim Morsli

**Affiliations:** Neurosurgical Department, BEO University Hospital, Algiers, Algeria E-mail: drkhelifaadel@gmail.com

**Keywords:** sarcoidosis, neurosarcoidosis, pineal region

## Abstract

Background: The central nervous system is an unusual location of sarcoidosis, which commonly affects the cranial nerves, meninges, hypothalamus, and pituitary gland. Involvement of the pineal region is extremely rare. This systematic review focused on the diagnosis and management of pineal region sarcoidosis, dorsal mesencephalon, and periaqueductal region.

Objectives: This study aimed to discuss diagnostic modalities and best management tools of the aforementioned pathology.

Methods: ScienceDirect, PubMed, and Google Scholar databases were searched for English or French articles about sarcoidosis of the pineal region, dorsal mesencephalon, and periaqueductal region. The clinical case of a patient managed at our department that we believe is directly relevant to this review is also presented. Patients’ demographics, clinical presentations, presence of hydrocephalus, other sarcoidosis locations in the central nervous system, and medical treatment were collected. Surgical management, surgical approach, and outcomes and complications of each procedure were also obtained. This study was conducted in agreement with the Preferred Reporting Items for Systematic Reviews and Meta-Analyses statement.

Results: Fifteen cases were examined. The study sample consisted of nine (60%) male and six (40%) female, and the mean age was 32 years. Eight (53%) patients had hydrocephalus, and the predominant clinical presentations were signs of increasing intracranial pressure (headaches, vomiting, and papilledema). Six (40%) patients had diplopia, and convergence–retraction nystagmus was noted in three (20%) patients. Argyll Robertson sign was present in one patient and suspected in another patient (13%). Medical treatment consisted mainly of steroids (93% of cases). Open surgery on the pineal region was performed in five patients, and four of them reported to have serious complications (such as ophthalmoplegia, hemianopsia, hemiparesis, bilateral third cranial nerve paresis, and cerebellar syndrome). Endoscopic management was performed in two patients without complications.

Conclusion: To treat hydrocephalus, brain imaging is mandatory in patients with sarcoidosis if intracranial hypertension is suspected. In pineal region sarcoidosis, management of hydrocephalus is the priority, followed by medical treatment of the lesion. Open surgery of any approach presents a high risk of complications; thus, an endoscopic approach is the preferred management, as it treats hydrocephalus and makes biopsy possible with minimal risk.

## Introduction

Sarcoidosis is a systemic condition of unknown origin^1,2^; neurosarcoidosis is relatively uncommon: usually located on the cranial nerves, meninges, hypothalamus, and pituitary gland.^2^ Pineal region involvement is extremely rare, and few cases were reported. Pineal region sarcoidosis (PRS) is challenging to diagnose and manage. This study is considered a wider update about PRS and thus aimed to clarify the best diagnosis and management methods for this pathology.

## Materials And Methods

### Case report

The patient was a 51-year-old female nurse with a medical history of high blood pressure, type I diabetes, and cholecystectomy. The patient was followed for systemic sarcoidosis under steroids, colchicine, and chloroquine. The patient presented with a sudden diplopia accompanied with intermittent headaches.

**Figure 1.**
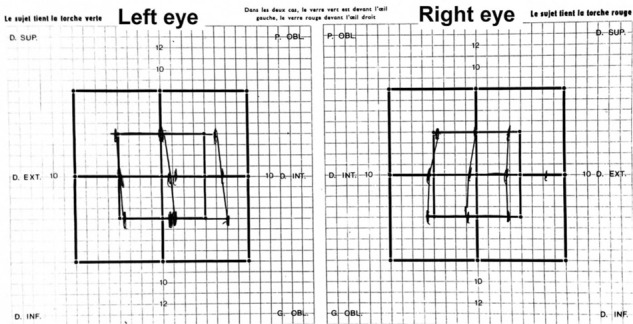
Preoperative Lancaster red-green test, attaching the diplopia to bilateral sixth cranial nerve palsy.

**Figure 2.**
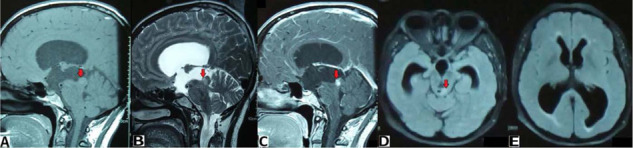
Preoperative brain MRI. (A) Sagittal tbl1 WI, (B) sagittal tbl2 WI, (C) sagittal tbl1 WI injected, (D) axial FLAIR passing through the Sylvius aqueduct, (E) axial FLAIR passing through the lateral ventricles’ frontal horns. The lesion in the tectum appears isointense on tbl1 WI and hyperintense on tbl2 WI and FLAIR images, with remarkable enhancement after gadolinium injection (arrow). The lesion caused an obstructive hydrocephalus (E).

Neurological examination did not find any objective deficit. The ophthalmological examination found bilateral sixth nerve palsy in the Lancaster test (Figure 1), her visual acuity was 4/10 and 5/10, and there was a stage I papilledema (bilateral hyperhemia). Initially, brain magnetic resonance imaging (MRI) on a 3-Tesla machine was performed, which objectified a lesion of 8 mm located on the quadrigeminal plate, isointense on tbl1-weighted imaging (WI), hyperintense on tbl2-WI and fluid-attenuated inversion recovery (FLAIR) sequences, with a nodular enhancement after gadolinium injection. This lesion was applying a pressure on the aqueduct of the Sylvius, causing obstructive, active triventricular dilation (Figure 2).

**Figure 3.**
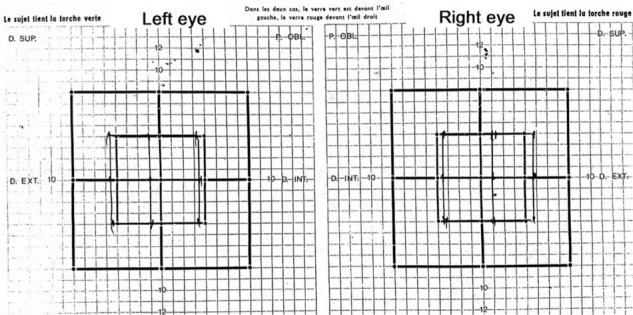
Postoperative Lancaster red-green test, showing disappearance of the diplopia.

**Figure 4.**
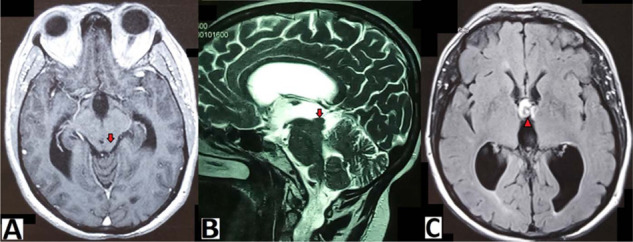
15 months postoperative MRI. (A) Axially injected tbl1 WI, (B) sagittal tbl2 WI, (C) axial FLAIR. The tectum lesion disappeared completely (arrow), with appearance of probable new location in the hypothalamus region (arrow head) and stabilization of the hydrocephalus.

An endoscopic third ventriculostomy and biopsy of the lesion were performed. Postoperatively, the patient's condition improved clinically and diplopia disappeared (Figure 3). The interval between headache episodes and the pain became tolerable, which appeared to have no relation with the previous hydrocephalus. The histological study found sites of tissue fragments with granulomatous lesions. These granulomas are composed of neutrophils, epithelioid cells, and lymphocytes. There was no caseating necrosis, so the diagnosis of neurosarcoidosis was retained. Postoperative medical treatment (steroids and colchicine) was continued. Brain MRI performed 7 months and 15 months later reveals total reduction of the quadrigeminal plate lesion and stabilization of the hydrocephalus. However, there was a suspected pituitary stalk and hypothalamus hypersignal on FLAIR sequences, but there was no neurological or endocrine correlation (Figure 4).

### Literature search

ScienceDirect, PubMed, and Google Scholar databases were searched for journal articles about sarcoidosis of the pineal region, dorsal mesencephalon, and periaqueductal region. The following keywords were used: “pineal,” “tectal,” “tectum,” “quadrigeminal,” “mesencephalon,” “aqueduct,” “splenium,” “tentorium,” “tentorial,” “obstructive hydrocephalus,” “noncommunicating hydrocephalus,” and “triventricular hydrocephalus.” Articles with title containing one of the keywords and “sarcoidosis” were reviewed. The study included English (n = 13) and French (n = 1) articles, and there was no publication date limitation. The Preferred Reporting Items for Systematic Reviews and Meta-Analyses guidelines were applied to this review.^3^ The protocol for this systematic review was not registered.

The following articles were excluded from the review: three articles in Japanese, three articles where the lesions were strictly located in the anterior mesencephalon, and one article where the full text was not available (abstract only without case presentation). Patients’ demographics, clinical presentations related to the pineal region location of the pathology, presence of hydrocephalus, other neurosarcoidosis locations, and medical treatment were collected. The study focused on the surgical management: whether surgery was performed, surgical approach, and outcomes and complications of each procedure. In one case report, the corresponding author was contacted (ophthalmologist) but could not give all previous data.

## Results

**Figure 5.**
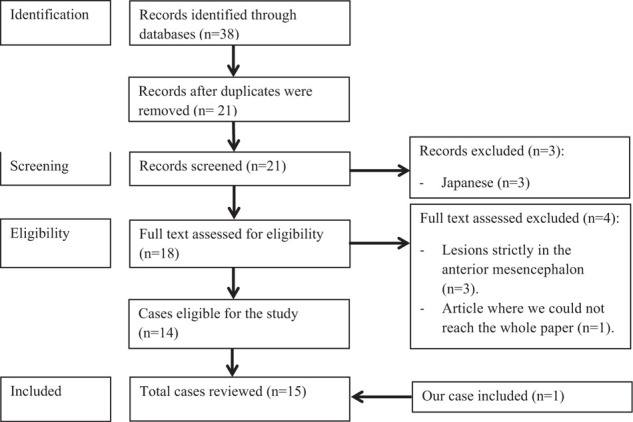
Flowchart according to the PRISMA statement.

The numbers of studies screened, assessed for eligibility, and included in the review are summarized in Figure 5. Including the present case, 15 papers were found to describe sarcoidosis of the pineal region, dorsal mesencephalon, and periaqueductal region. These cases are summarized in Table 1. The study sample consisted of nine (60%) male and six (40%) female patients. The mean age was 32 (range, 13–58) years. Eight (53%) patients had hydrocephalus; the main clinical presentations were signs of increasing intracranial pressure (such as headaches, vomiting, and papilledema). Six (40%) patients had diplopia, and convergence–retraction nystagmus was noted in three (20%) patients. Argyll Robertson sign was found in one patient and suspected in another patient (13%). Other presentations are related to other locations (Table 1). Medical treatment consisted of steroids in 14 (93%) cases. In one case, no pharmacological treatment was given after total removal of the lesion. Open surgery on the pineal region was performed in five patients; four of them reported serious complications, including ophthalmoplegia, hemianopsia, hemiparesis, bilateral third cranial nerve paresis, and cerebellar syndrome. Endoscopic management was performed in two patients without complications.

## Discussion

**Figure 6.**
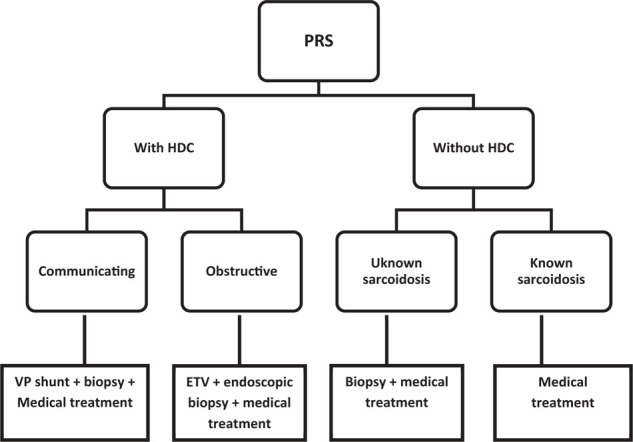
Our center's management protocol of pineal region sarcoidosis. PRS, pineal region sarcoidosis; VP, ventriculoperitoneal; ETV, endoscopic third ventriculostmy; HDC, hydrocephalus.

The pineal region is the part of the brain limited superiorly by the splenium, anteriorly by the posterior wall of the third ventricle, inferiorly by the tectal plate, laterally by the thalamus, and posteriorly by the falcotentorial angle, splenium, and vermis. Sarcoidosis is a systemic granulomatosis of unknown origin.^1,2^ Many authors have suggested that sarcoidosis granuloma is a result of an exaggerated immunologic response to an unknown antigen, causing inappropriate T lymphocyte reaction after acquiring a cellular immunity. This inappropriate response could have a genetic origin; in fact, many cases of familial sarcoidosis have been reported, but a causative gene has not been identified.^1,2^ An environmental influence has been suggested, as the pathology commonly affects some professionals such as firefighters and nurses.^1,2^ Nervous system involvement accounted for 5%–13% of all sarcoidosis cases^1,2,14^; cranial nerves, meninges, hypothalamus, pituitary gland, spinal cord, and peripheral nerves are commonly affected.^2,14^ Pineal region involvement is extremely rare.^4,5,10,12,13,14,16^ Similar to other neurosarcoidosis, PRS affects young adults^1,2^; only two patients in the present series were over 50 years old (age range, 13–58 years). Although neurosarcoidosis is more common in the female population according to most studies,^1,2^ pineal region involvement is more common in the male population (9 males/6 female). In 2019, Saban et al., reported 20 cases of hydrocephalus caused by neurosarcoidosis, and 60% presented with obstructive hydrocephalus.^18^ This case was also included in the present review; in fact, the major feature of PRS is the position of the lesion in the outflow of the cerebrospinal fluid (CSF), which could cause obstructive ventricular dilation. Hydrocephalus in neurosarcoidosis could also be nonobstructive (communicating); in this case, it is caused by granulomatous leptomeningitis in basal cisterns that reduces absorption of the CSF in the arachnoidal villi.^8,22,23^ Including our case, eight cases from the presented series had hydrocephalus, which is a serious complication. On forensic papers, several cases of sudden death were considered caused by obstructive hydrocephalus in neurosarcoidosis.^19-21^ Thus, brain imaging should be a routine, and conservative management of hydrocephalus should not be considered. In the present review, the clinical presentation of PRS was dominated by signs of intracranial hypertension in patients with active hydrocephalus, presented mainly with headaches, vomiting, and papilledema. Moreover, diplopia is very common; including our case, six (40%) patients had diplopia caused by sixth nerve palsy (Figure 1, Lancaster red-green test), which appears to be another presentation of intracranial hypertension. Two other signs were less common but more specific: convergence–retraction nystagmus was noted in three (20%) patients, and Argyll Robertson sign,^6^ was present in one case and suspected in another (13%). Other presentations are related to other lesion locations (Table 1). Brain computed tomography (CT) is important in diagnosing hydrocephalus and its type (communicating or obstructive), which indicates the need for emergent management. Before the era of MRI, contrast CT was the preferred examination: it can detect nodular enhancement in the pineal region. MRI is now the study of choice. In this review, PRS was detected by MRI in 11 cases, including our case; it was isointense on tbl1-WI and hyperintense on tbl2-WI and FLAIR sequences, with remarkable nodular enhancement after gadolinium injection in all cases. Laboratory tests are not always helpful in diagnosis; elevated levels of serum angiotensin-converting enzyme (ACE) are seen in up to 65% of patients, and levels of ACE in the CSF are also elevated in 55% of the patients. CSF tests may be performed for lymphocytic pleocytosis and elevated protein levels, but the glucose level may be decreased.^1,2^ In 1999 Zajicek et al., proposed three levels of diagnosis probability for neurosarcoidosis: definite, probable, and possible.^24^ In definite neurosarcoidosis, nervous system non-caseating granulomas were found in the biopsy, with central nervous system (CNS) clinical syndrome and exclusion of other origins. The diagnosis is probable if the patient presents a clinical syndrome involving sarcoidosis, with other examinations supporting the diagnosis, such as MRI or laboratory tests suggest a CNS inflammation (elevated levels of CSF proteins or cells, presence of oligoclonal bands) as well as positive systemic sarcoidosis (either positive histology or at least two indirect indicators from Gallium scan, chest imaging, and serum ACE), with no evidence of alternative disease. Finally, the diagnosis is possible if a clinical syndrome is present, lesions from other origins were not found, and previous criteria are not met.^2,24^ Our PRS management depends on whether the diagnosis of sarcoidosis is already made and on the presence or absence of hydrocephalus and its type (Figure 6). If no ventricular dilation was present, medical treatment could be efficient if the diagnosis is highly suspected, but if other diagnosis options fail, a biopsy is indicated. In case of communicating hydrocephalus, ventriculoperitoneal (VP) shunt was the best option and provided good results.^18^ In case of obstructive hydrocephalus, endoscopy appears to be the treatment of choice, as it offers multiple advantages in a one-step surgical approach: first draining the hydrocephalus by endoscopic third ventriculostomy (ETV), followed by measurement of tumor markers and cytological analysis of the CSF. The surgeon could also perform a biopsy in visu of the lesion. Finally, shunt-related complications are not possible.^25^ In the present series, most patients who underwent open surgery experienced complications postoperatively. The most serious complications related to open surgery are oculomotor deficit,^4,9-11^ hemiparesis,^9^ cerebellar syndrome,^9^ cephalic tremor,^9^ and hemianopsia.^11^ Our case and another case in the review were managed by endoscopic biopsy and ETV, which both remarkably improved postoperatively. An external ventricular drain appears to be not a good choice anymore; it gives a temporary solution for the hydrocephalus and increases the patient's risk of meningitis. Herein, it was used in two patients: replaced in one by a VP shunt^15^ and removed in the other case on postoperative day 7 without need for any shunt.^10^ In this review, PRS is often accompanied with lesions located in other parts of the CNS (40%); thus, the physician should be aware and predict those locations before the onset of major signs. In PRS management, complications are related to invasive procedure aiming at aggressive removal; otherwise, the outcome is generally good with total recovery of most patients, and the prognosis is related to other systemic complications of the disease.

## Conclusion

Brain imaging is mandatory in patients with sarcoidosis if intracranial hypertension is suspected to eliminate the presence of hydrocephalus. In case of PRS, the management of hydrocephalus is the priority, followed by medical treatment. If diagnosis is already known, medical treatment could be totally efficient. Biopsy is indicated in the absence of clear diagnosis. Open surgery of any approach presents a high risk of complications; thus, endoscopy is the preferred management, as it treats the hydrocephalus and makes biopsy possible with minimal risk.

## Figures and Tables

**Table 1:** Summary of reviewed articles about PRS.

Cases	Age	Sex	Clinic	HDC	Other CNS locations	Pharmacotherapy	Surgical management	Outcome & complications

Schaefer, et al. 1977. [4]	13	M	Papilledema, Nystagmus, positive Babinski EEG altered.	Yes	None.	None.	Ventriculoatrial shunt. Removal through sub-occipital trans-tentorial approach.	Transient vertical visual paresis and a transient psycho-organic syndrome.

Giroud, et al. 1983. [5]	40	F	Optic atrophy.	Yes	None.	Steroids.	Ventriculoatrial shunt.	Improvement.

Poole, et al. 1984. [6]	28	F	cranial polyneuropathy, Argyll Robertson pupils.	No	None.	Steroids.	None.	?

Wall, et al. 1985. [7]	32	M	Seizure, mental deterioration, pan-hypopituitarism.	NO	Suprasellar, basal ganglia, brain stem spinal cord.	Steroids.	None.	Improvement, then dead from aspiration during seizure.

Sherman, et al. 1987. [8]	34	F	Neuro-endocrine dysfunction.	No	Leptomeningeal diffuse.	Steroids.	None.	?

Martin, et al. 1989. [9]	18	M	Headache, vomiting, diplopia, nystagmus.	Yes	None.	Steroids.	Shunt. Open biopsy.	Cerebellar syndrome, hemiparesis, cephalic tremor, persistence of neuro-ophthalmic signs.

Sattelmeyer, et al. 1999. [10]	26	M	Intermittent headache, vomiting, papilledema, homonymous hemianopsia.	Yes	None.	Steroids.	External ventricular drain removed day 7 post-operatives. Sub-totally removed through an occipital trans-tentorial approach.	Bilateral third cranial nerve paresis, and ophthalmoplegia.

Klainguti, et al. 2004. [11]	26	M	Headaches, Intermittent hemineglect..	?	?	Steroids.	Biopsy (approach?).	Homonymous hemianopsia, bilateral third cranial nerve paresis, and ophthalmoplegia.

Oichi, et al. 2008. [12]	29	M	Diplopia, nystagmus, photomotor reflex altered. (Argyll Robertson?).	No	None.	Steroids.	None.	Improvement.

Westhout, et al. 2008. [13]	40	M	Headache, papilledema, diplopia.	Yes	Medial temporal lobe, posterior limbs of the internal external and extreme capsules bilaterally.	Steroids.	VP-shunt. Biopsy of the temporal lesion.	Improvement.

Yang, et al. 2009.[14]	45	M	Intermittent headaches, blurry vision, diplopia, nausea, coordination difficulties.	No	None.	Steroids.	Biopsy through supra cerebellar infra-tentorial approach.	Improvement.

Chandna, et al. 2015. [15]	41	F	Headache, papilledema, diplopia, gait disturbance, hemibody weakness.	Yes	Leptomeningeal diffuse.	Steroids.	External ventricular drain. VP-shunt.	Improvement, then death from pulmonary emboli.

Nakayasu, et al. 2019. [16]	58	F	Gait disturbance, dizziness.	Yes	None.	Steroid only for a skin lesion.	ETV and endoscopic biopsy.	Improvement.

O'Connor, et al. 2019. [17]	47	M	Hypopituitarism.	None	Pituitary gland, optic chiasm, hypothalamus.	Steroids.	None.	Improvement.

This report	51	F	Intermittent headaches, ↓ ↓ visual acuity, papilledema, diplopia (VI nerve palsy).	Yes	Hypothalamus.	Steroids, colchicine and chloroquine.	ETV and endoscopic biopsy.	Improvement.


PRS: pineal region sarcoidosis; VP: ventriculo-peritoneal; ETV: endoscopic third ventriculostomy; CNS: central nervous system; HDC: hydrocephalus.
